# The Role of Antioxidant Compounds from Citrus Waste in Modulating Neuroinflammation: A Sustainable Solution

**DOI:** 10.3390/antiox14050581

**Published:** 2025-05-11

**Authors:** Alessia Silla, Angela Punzo, Cristiana Caliceti, Maria Cristina Barbalace, Silvana Hrelia, Marco Malaguti

**Affiliations:** 1 Department of Biomedical and Neuromotor Sciences, Alma Mater Studiorum, University of Bologna, 40126 Bologna, Italy; alessia.silla2@unibo.it (A.S.); angela.punzo2@unibo.it (A.P.); cristiana.caliceti@unibo.it (C.C.); 2 Department for Life Quality Studies, Alma Mater Studiorum, University of Bologna, 47921 Rimini, Italy; maria.barbalace2@unibo.it (M.C.B.); marco.malaguti@unibo.it (M.M.)

**Keywords:** citrus waste, polyphenols, neuroinflammation, neurodegeneration, antioxidant activity, bioavailability, oxidative stress, sustainability

## Abstract

In normal conditions, neuroinflammation induces microglia and astrocyte activation to maintain brain homeostasis. However, excessive or prolonged neuroinflammation can inflict harmful damage on brain tissue. Numerous factors can trigger chronic neuroinflammation, ultimately leading to neurodegeneration. In this context, considering the pressing need for novel, natural approaches to mitigate neuroinflammatory damage, attention has turned to unconventional sources such as agricultural by-products. Citrus fruits are widely consumed globally, producing substantial waste, including peels, seeds, and pulp. Traditionally regarded as agricultural waste, these by-products are now recognized as valuable reservoirs of bioactive compounds, including flavonoids, carotenoids, terpenoids, and limonoids. Among these, citrus polyphenols—particularly flavanones like hesperidin, naringenin, and eriocitrin—have emerged as potent modulators of neuroinflammatory pathways through their multifaceted interactions with cellular antioxidant systems, pro-inflammatory signaling cascades, neurovascular integrity, and gut–brain axis dynamics. This review aims to characterize the key molecules present in citrus waste and synthesizes preclinical and clinical evidence to elucidate the biochemical mechanisms underlying neuroinflammation in neurodegenerative disorders.

## 1. Introduction

Neuroinflammation, together with oxidative stress, protein aggregation and misfolding, mitochondrial dysfunctions, and other factors, is a common thread in the development and progression of many neurodegenerative diseases [1,2]. Recently, increasing attention has been directed toward natural compounds as potential therapeutic agents able to counteract neurodegeneration at multiple levels [1]. In this framework, agrifood by-products have emerged as innovative and sustainable reservoirs of natural bioactive compounds with potential health benefits, including the prevention and mitigation of chronic diseases [3]. Notably, agrifood by-products represent one of the most significant sources of global waste, significantly contributing to greenhouse gas emissions and environmental degradation [4]. Thus, this challenge has driven growing interest in valorizing these secondary streams.

Recent advancements in green chemistry have enabled the efficient recovery of antioxidant and bioactive compounds from various agrifood waste, including seeds, peels, leaves, bran, kernels, and pomace [5,6]. Several studies have reported the possible neuroprotective effects of plant-based agrifood waste derived from widely produced and consumed products, including grapes, wine, coffee, tomatoes, olive oil, chestnuts, onions, apples, and pomegranates [1].

This review examines the potential antioxidant and anti-neuroinflammatory properties of bioactive compounds derived from the waste and by-products of the Citrus genus, a fruit crop widely cultivated globally.

## 2. Literature Search Strategy

A literature search was conducted to identify studies investigating the relationship between the Citrus genus and its effects on neuroinflammation and oxidative stress.

Searches were performed in PubMed, Scopus, and Web of Science (Core collection). The inclusion criteria covered studies published between 2015 and 2025. However, exceptions were permitted for earlier publications that are extensively cited and offer fundamental insights into the chemical profile of citrus waste and the bioavailability of citrus-derived phytochemicals (e.g., [7,8]).

The search strategy combined the descriptors using the Boolean operators (AND/OR) as follows: citrus AND (waste OR “by-products”) AND (neuro* OR inflammation OR “oxidative stress”). A total of 259 papers were retrieved. Only peer-reviewed articles written in English and available in full text were included. Since not all the articles precisely addressed the topics covered by the review, a selection process was independently carried out by the six authors, resolving potential conflicts through confrontation and discussion.

**Figure 1:**
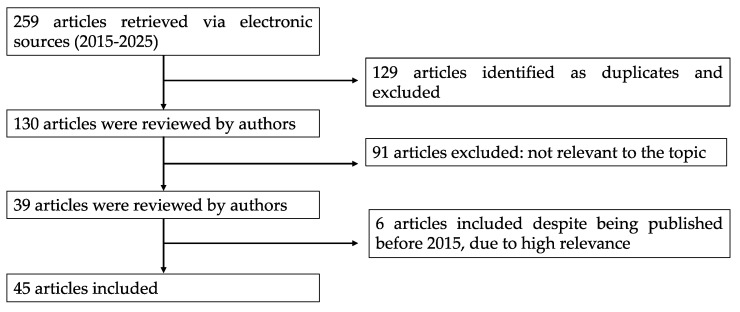
Flow chart of the bibliographic search strategy.

The results of the search and screening activities are summarized in the flowchart in Figure 1.

## 3. Molecular Mechanisms in Neuroinflammation

Inflammation serves as a primary immune defense mechanism against infections, trauma, toxin buildup, and other pathological insults [9,10]. In the central nervous system (CNS), neuroinflammation represents a complex and multifaceted specialized inflammatory response. It involves several cell types, including microglia, astrocytes, and oligodendrocytes, that work together in a coordinated and synergistic manner via neurotransmitters, ions, neurotrophic factors, and cytokines [11]. Among these, microglia and astrocytes play pivotal roles in mediating the neuroinflammatory response [10].

Under normal circumstances, microglia function similarly to macrophages by maintaining brain homeostasis and undertaking various reparative tasks. These include clearing dendritic debris, organizing synapses, responding to biotoxins, and phagocytosing abnormal proteins [12]. For example, during the development of Alzheimer’s disease (AD), microglia can help safeguard the brain against neurodegeneration by clearing β-amyloid (Aβ) [13,14]. Additionally, activated microglia stimulate astrocyte proliferation, thereby promoting neuroprotection and the repair of damaged neural tissue [15,16]. When astrocytes become activated, they exhibit morphological and biochemical changes, such as cell hypertrophy, increased regulation of intermediate filaments, and enhanced proliferation and mobility in response to brain injury [17].

Despite its protective role, excessive or prolonged neuroinflammation can cause significant damage to brain tissue. [18,19]. Factors like aging, metabolic disorders, and viral infections may trigger chronic neuroinflammatory states, ultimately leading to neurodegeneration [20]. The impairment of pro-survival pathways, such as autophagy, might also be responsible for neuroinflammation. Impaired autophagy contributes to chronic microglial activation and the release of pro-inflammatory cytokines, and this mechanism is implicated in the pathogenesis of several neurodegenerative diseases [21,22].

A key feature of neuroinflammation is the activation of Nuclear Factor Kappa B (NF-κB), a transcription factor that orchestrates the expression of various genes implicated in inflammation, apoptotic cell death, cell survival, and neuronal differentiation [23].

Microglial activation is generally classified into two phenotypes: the classical M1, which exerts pro-inflammatory effects, and the alternative M2, which is anti-inflammatory, mirroring the paradigm observed in macrophages [24,25,26]. The simultaneous engagement of different signaling pathways, mediated by Toll-like receptors (TLRs) and interferon-γ (IFN-γ), drives the development of the M1 phenotype [27,28]. M1 microglia are characterized by the production of pro-inflammatory cytokines and chemokines that contribute to tissue damage [28]. Via NF-κB activation, these cells upregulate inflammatory genes such as inducible nitric oxide synthase (iNOS) and cyclooxygenase-2 (COX-2), leading to the release of mediators like tumor necrosis factor (TNF)-α, interleukin (IL)-6, IL-1, reactive oxygen species (ROS), and β-secretase1 (BACE1) [29,30]. Furthermore, M1 microglia overexpress enzymes like NADPH oxidase and iNOS, resulting in increased production of superoxide anions while concurrently exhibiting reduced phagocytic activity [31,32]. This phenotype can adversely affect the differentiation and maturation of neural progenitor cells, synapse formation, and overall neural plasticity.

Conversely, the neuroprotective M2 phenotype can be induced by cytokines such as IL-4, IL-10, IL-13, and transforming growth factor-β (TGF-β). These signals promote the release of various factors, including FIZZ1, chitinase-3-like-3 (Chi3l3), Arginase 1, Ym1, CD206, insulin-like growth factor 1 (IGF-1), and frizzled class receptor 1 (Fzd1), which are associated with an enhanced phagocytic capacity, improved synaptic communication, increased plasticity, and neuronal repair [33,34]. For example, IL-4 has been shown to inhibit the secretion of pro-inflammatory cytokines such as IL-6, TNF-α, and nitric oxide (NO) [35].

**Figure 2:**
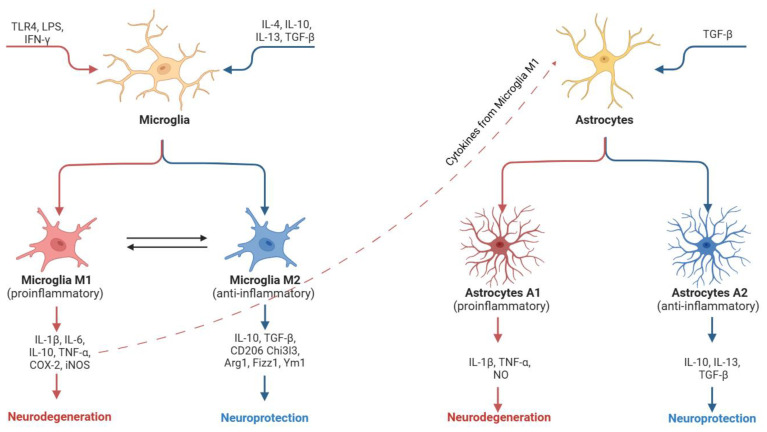
Schematic representation of microglia and astrocyte activation. Neuroinflammatory stimuli, such as those mediated by Toll-like receptors (TLRs) and interferon-γ (IFN-γ), promote polarization into M1 microglia. M1 microglia, by releasing pro-inflammatory cytokines, trigger astrocyte polarization into the A1 phenotype, further promoting neurodegeneration. Cytokines such as interleukin-4 (IL-4), interleukin-10 (IL-10), interleukin-13 (IL-13), and TGF-β promote M2 microglia and A2 astrocyte polarization, which are responsible for neuroprotection. The figure is original and created with BioRender.com (accessed on 15 April 2025).

Reactive astrocytes have been similarly categorized into two phenotypes: A1 and A2 [36,37]. A1 astrocytes, which are activated by cytokines released from M1 microglia, lose many of their normal functions, such as maintaining synaptic integrity, and may secrete soluble neurotoxins that rapidly kill certain neurons and mature oligodendrocytes [36,38]. Specifically, A1 astrocytes increase the expression of genes (e.g., those in the complement cascade) and release pro-inflammatory factors, including IL-1β, TNF-α, and NO, thereby exacerbating neuroinflammation [38]. In contrast, the A2 phenotype is promoted by TGF-β [39] and appears to be neuroprotective, as it promotes the synthesis of neurotrophic and anti-inflammatory agents that support neuronal survival, growth, and repair [38]. Once activated, A2 can subsequently release factors like IL-4, IL-10, and TGF-β [40] (Figure 2).

While the binary classification of M1/M2 microglia and A1/A2 astrocytes simplifies the understanding of these cell roles, it is recognized that activated microglia and reactive astrocytes can also exhibit mixed or intermediate phenotypes [41,42]. Nevertheless, this framework provides valuable insight into the reactive states of these cells in various CNS disorders.

Recent advances have significantly expanded our understanding of neuroinflammation, revealing several novel mechanisms that complement traditional paradigms. One notable discovery is the role of the NLRP3 inflammasome as a critical regulator of neuroinflammatory responses that participate in the development of the M1 microglia phenotype. Its activation has been closely linked with the pathogenesis of neurodegenerative diseases such as Parkinson’s disease (PD) and AD, making it a promising target for therapeutic intervention [43]. Concurrently, research into epigenetic regulation has uncovered that DNA methylation and histone modifications, along with non-coding RNAs, including microRNAs and long non-coding RNAs, can dramatically influence the expression of inflammatory genes in glial cells. These molecular alterations modulate microglial and astrocytic activities, thereby affecting the overall neuroinflammatory state [44].

Another emerging area is the gut–brain axis, which underscores the bidirectional communication between the intestinal microbiota and the central nervous system. Signals derived from gut bacteria, transmitted via immune, neural, and endocrine pathways, can modulate glial activation and influence the progression of CNS disorders [45].

In summary, neuroinflammation is a major pathogenic factor in numerous neurodegenerative diseases, including PD, AD, and amyotrophic lateral sclerosis (ALS). Therefore, it represents a critical target for therapeutic intervention to mitigate neurodegeneration [46].

## 4. Citrus Waste as a Source of Antioxidant Bioactive Compounds

The *Citrus* genus, belonging to the *Rutaceae* family, comprises some of the world’s most extensively cultivated fruit crops, with annual production exceeding 158 million tons [47]. Among these, the most widely produced crops include sweet oranges (*C. sinensis*), mandarins (*C. reticulata*, *C. tangerine*, *C. clementina*), lemons (*C. limon*), bergamots (*C. bergamia*), and pomelo (*C. grandis*) [48], supporting a global industry with their distinctive organoleptic properties, nutritional benefits, and wide range of applications.

Approximately 40% of global citrus production undergoes industrial processing, primarily for juice extraction, resulting in substantial amounts of organic by-products, including peels, seeds, and leftover pulp. These by-products account for 50–70% of the original fruit mass, resulting in an estimated annual accumulation of over 40 million tons of waste [49]. Disposing of this biomass poses significant environmental and economic challenges due to its high organic load and potential for soil and water contamination.

**Figure 3:**
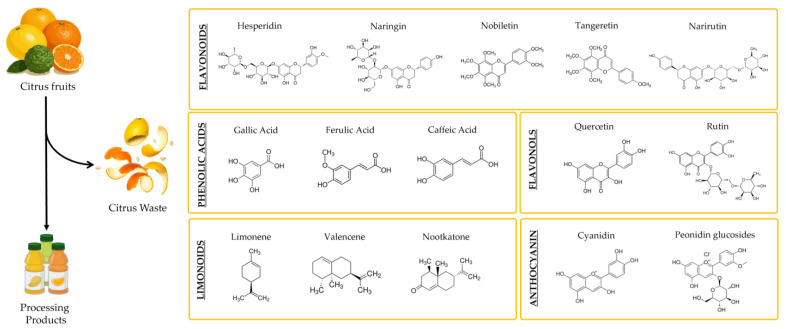
Main phytochemicals present in citrus waste. The figure is original and created with BioRender.com (accessed on 9 April 2025).

Citrus by-products are rich in various chemical compounds, including free sugars (e.g., glucose, fructose, sucrose), organic acids (citric, malic, malonic, oxalic), carbohydrate polymers (cellulose, hemicellulose, pectin), enzymes (pectinesterase, phosphatase, peroxidase), essential oils, terpenoids, and pigments [50]. Notably, citrus peels and seeds are an important source of bioactive compounds [51] with recognized health-promoting properties, including antioxidant, anti-inflammatory, and antimicrobial activities [52,53]. The main bioactive molecules found in citrus waste are summarized in Figure 3.

Among these, flavonoids, specifically 7-O-glycosyl flavanones, represent the most abundant compounds identified in citrus by-products [54]. Flavonoids typically occur as glycosides or their corresponding aglycones. Key aglycones include naringenin and hesperetin, while glycosides are broadly categorized into neohesperidosides and rutinosides [55]. Neohesperidoside flavanones (e.g., naringin, neohesperidin, neoeriocitrin) are composed of a flavanone linked to neohesperidose (rhamnosyl-α-1,2-glucose) and are characterized by their bitter taste. Conversely, rutinoside flavanones (e.g., hesperidin, narirutin, didymin) are characterized by a flavanone linked to rutinose (rhamnosyl-α-1,6-glucose) and are typically tasteless. Some species, such as sweet orange and mandarin, mainly accumulate rutinosides, while others, such as pomelo, are rich in neohesperidoside flavanones [55].

Citrus waste additionally encompasses polymethoxylated flavones (PMFs) (e.g., nobiletin, tangeritin, and 5-dimethyl nobiletin) [56], phenolic acids (e.g., gallic, chlorogenic, and caffeic acids) [57,58], flavonols (e.g., quercetin, rutin, and kaempferol) [59], and anthocyanins (cyanidin and peonidin glucosides), all of which are well-documented for reducing inflammation and oxidative stress [60].

In addition to polyphenol compounds, citrus by-products also contain limonoids, a subclass of triterpenoids including limonene, valencene, and nootkatone [61]. These compounds contribute to the characteristic aroma of citrus fruits and exhibit several biological properties, including antioxidant activity [62].

Species, cultivars, and tissue types significantly affect the composition of these bioactive compounds. For instance, the peels and seeds of *C. reticulata* are rich in hesperidin, narirutin, and naringin, as well as significant concentrations of PMFs, including nobiletin and tangeretin [63,64]. In *C. limon*, the peel primarily accumulates neoeriocitrin and neohesperidin [7], whereas the seeds exhibit a substantial concentration of eriocitrin [65]. In *C. sinensis*, peels and seeds are high in hesperidin, naringin, and narirutin [7,8].

The chemical profile of citrus waste, particularly its high flavonoid content, offers a sustainable opportunity to harness health-promoting compounds. These phytochemicals have shown promising effects on cellular pathways related to inflammation and oxidative stress, creating opportunities for addressing inflammation-related diseases, including those affecting the CNS. Therefore, recovering these bioactive molecules from citrus by-products promotes environmental sustainability and paves the way for their application in nutraceutical and therapeutic fields.

### Bioavailability of Bioactive Antioxidant Compounds in Citrus Waste

Despite their therapeutic potential, the efficacy of citrus-derived bioactive compounds is limited by poor bioavailability [66,67].

Flavonoids exhibit low water solubility and a large molecular size, restricting their dissolution and gastrointestinal absorption [68]. Furthermore, they undergo substantial phase I metabolism (oxidation, reduction, hydrolysis), followed by phase II conjugation reactions (glucuronidation, sulfation, methylation) mediated by UDP-glucuronosyltransferases (UGTs) and sulfotransferases (SULTs), in hepatic and intestinal cells [69]. Additionally, gut microbiota can metabolize flavonoids into phenolic and aromatic ring-fission metabolites, which may alter their bioactivity and absorption [70]. For instance, naringenin is metabolized in the lower intestine by *Streptococcus S-2*, *Lactobacillus L-2*, and *Bacteroides JY-6*, generating a series of low-molecular-weight aromatic acids [71]

Evidence indicates that colon bacteria play a central role in hydrolyzing flavanone glycosides—such as hesperidin and naringin—into their aglycones, hesperetin, and naringenin, which are subsequently absorbed systemically [72]. These glycosides, both rutinosides, are not hydrolyzed by β-glucosidases in the small intestine but are instead hydrolyzed in the distal intestine and colon by the enteric microflora [73]. The hydrolysis step seems to be the rate-limiting factor for absorption. In contrast, flavonoid aglycones are absorbed efficiently in the small intestine, which explains the faster kinetics [74]. For instance, through pharmacokinetic analysis in human subjects, Kanaze et al. have shown that both hesperetin and naringenin were rapidly absorbed, and their concentrations in plasma were observed 20 min after dosing and reached a peak in 4.0 and 3.5 h, respectively [74].

Citrus limonoids face additional challenges regarding bioavailability. Their rigid triterpenoid structure resists enzymatic cleavage, and even at high oral doses, plasma levels remain minimal [75]. Additionally, hepatic metabolism via CYP2D6 and CYP3A4 contributes to their rapid clearance, although some limonoid metabolites have been detected systemically [75].

Following absorption and metabolic processes, the ability of bioactive antioxidant compounds to exert effects within the central nervous system is contingent upon their capacity to cross the blood–brain barrier (BBB). Preclinical models have shown that flavonoid aglycones (e.g., hesperetin, naringenin) and their significant metabolites can pass through the BBB through passive diffusion [76,77]. On the other hand, glycosylated forms (e.g., naringin, neoeriocitrin) exhibit limited BBB permeability [78] without microbial biotransformation, leading to substantial inter-individual variability. Human data concerning the brain bioavailability of other citrus compounds, such as polyphenol metabolites and limonoids, are lacking, hindering the translation of preclinical findings to neuroinflammation therapeutics.

Strategies to enhance bioavailability include nanoencapsulation [79] and lipid-based delivery systems [80], which improve aqueous solubility, intestinal uptake, and metabolic stability. Addressing these limitations through targeted research is essential to unlock the therapeutic potential of citrus bioactive compounds.

In conclusion, citrus waste represents a rich and underutilized source of bioactive compounds, particularly flavonoids, which exhibit promising beneficial properties, including antioxidant and anti-inflammatory activities. While bioavailability and BBB permeability remain limiting factors, especially in clinical contexts, growing preclinical data support their potential role in targeting neuroinflammatory mechanisms.

The following section will discuss the impact of citrus-derived compounds, particularly those extracted from industrial by-products, on the fundamental mechanisms associated with neuroinflammatory conditions.

## 5. Citrus By-Products and Neuroinflammation: Mechanisms and Potential Applications

Traditionally discarded as agricultural waste, citrus peels, seeds, and pulp are now recognized as sustainable sources of neuroprotective agents that exhibit potent anti-inflammatory, antioxidant, and beneficial properties, with potential applications in nutraceuticals and functional foods [81,82]. Recent research has increasingly highlighted that these by-products are rich in flavonoids, terpenoids, and limonoids, which play crucial roles in modulating neuroinflammation and oxidative stress, key pathological mechanisms underlying neurodegenerative disorders such as AD and PD [76,83].

Studies on various citrus polyphenols from different species, including *C. reticulata*, *C. sinensis*, *C. limon*, *C. grandis*, and *C. bergamia*, have demonstrated their ability to mitigate neuroinflammation through the inhibition of key pro-inflammatory mediators such as TNF-α, IL-6, iNOS, COX-2, and NF-κB [84,85,86,87]. By preventing IκBα phosphorylation, flavonoids such as rutin, naringin, and hesperetin block NF-κB translocation, thereby reducing inflammatory cytokine production and microglial activation [88,89,90]. Additionally, citrus polyphenols, including hesperitin, naringenin, and nobiletin, exert their effects by downregulating inflammatory cytokine expression, scavenging ROS and nitrogen (RNS) species, and modulating crucial intracellular pathways such as the phosphoinositide 3-kinase/protein kinase B (PI3K/Akt) cascade and mitogen-activated protein kinase/extracellular signal-regulated kinase (MAPK/ERK) pathway, both of which play vital roles in neuronal survival and stress responses [91,92,93,94]. Moreover, citrus-derived bioactive compounds such as hesperidin, naringenin, and eriocitrin exhibit significant neuroprotective effects by modulating autophagy pathways in the nervous system [95,96]. These flavonoids act through mechanisms including activation of AMPK and inhibition of mTOR signaling, thereby promoting autophagic flux and cellular clearance [97,98]. Hesperidin has been shown to modulate autophagic balance and reduce neuroinflammation in neurodegenerative models [96,99]. Naringenin enhances neuronal survival by modulating the expression of autophagic genes (Beclin-1 and LC-3) and mitigating oxidative stress [100].

Clinical trials have shown that supplementation with citrus extracts significantly reduces plasma IL-6 and C-reactive protein (CRP) levels, supporting their anti-inflammatory potential in humans [101,102]. Furthermore, metabolomic and transcriptomic studies have provided deeper insight into the mechanisms by which citrus-derived compounds support neuronal function [103]. Tangerine peel extract, for instance, has been shown to exert anti-neuroinflammatory effects through a synergistic interaction between flavonoids such as hesperidin, nobiletin, and tangeretin, which together significantly reduce NO production and inflammatory damage [104]. Similarly, orange by-products obtained through pressurized liquid extraction (PLE100) have been found to contain over 450 bioactive compounds that enhance synaptic plasticity and protect dopaminergic neurons, reducing Aβ-induced toxicity in AD models [84]. Hesperetin, a metabolite of hesperidin, has been shown to increase the expression of heme oxygenase-1 (HO-1) by 40–60% in dopaminergic neurons, reducing ROS levels and preventing neuronal apoptosis in PD models [95]. Indeed, citrus-derived polyphenols and terpenoids also exhibit significant anti-cholinesterase activity, inhibiting key enzymes such as acetylcholinesterase (AChE) and butyrylcholinesterase (BuChE), which are implicated in the breakdown of neurotransmitters like acetylcholine. This enzymatic inhibition plays a crucial role in preserving cholinergic signaling, thereby improving cognitive function and mitigating neurodegeneration [61,85].

Citrus polyphenols also exert potent antioxidant effects through activation of the nuclear factor erythroid 2-related factor 2 (Nrf2)–antioxidant response element (ARE) pathway (Nrf2/ARE), a critical cellular defense mechanism against oxidative stress. By dissociating Nrf2 from its cytoplasmic inhibitor Keap1, these compounds facilitate its nuclear translocation, inducing the expression of antioxidant enzymes such as superoxide dismutase (SOD), glutathione peroxidase (GPx), and HO-1 [105,106,107]. Moreover, Nrf2 activation enhances mitochondrial biogenesis via peroxisome proliferator-activated receptor gamma coactivator 1-alpha (PGC-1α), restoring the activity of electron transport chain (ETC) complexes I–IV, which are often impaired in neurodegenerative conditions [95,108]. These effects collectively counteract oxidative damage implicated in AD, PD, and ischemic stroke [109,110].

Over 90% of citrus flavanones undergo microbial metabolism, yielding bioactive derivatives such as 3-hydroxyphenylpropionic acid, which enhances short-chain fatty acid (SCFA) production (e.g., butyrate) and reduces systemic inflammation [111,112]. These microbial metabolites play a central role in gut–brain axis communication, modulating neuroinflammation through multiple interconnected mechanisms. Indeed, SCFAs such as butyrate can activate G-protein-coupled receptors (e.g., GPR41, GPR43) [113], inhibit histone deacetylases (HDACs) [114], and promote regulatory T-cell function [115], all contributing to a reduction in systemic and central inflammation. Moreover, citrus polyphenols indirectly support the integrity of the gut barrier, limiting the translocation of lipopolysaccharides (LPSs) into the circulation, a key trigger of microglial activation [116]. The resulting decrease in circulating pro-inflammatory mediators (e.g., IL-6, TNF-α) contributes to the attenuation of neuroinflammatory signaling pathways such as NF-κB and MAPK in the brain [117]. Studies indicate that citrus consumption increases *Bifidobacterium* spp. abundance by 40%, correlating with lower glial fibrillary acidic protein (GFAP) expression and reduced NF-κB activity in the brain [118,119]. These findings highlight the role of gut microbiota in amplifying polyphenol efficacy through metabolite generation and immune modulation, further emphasizing the importance of dietary interventions in neuroprotection [120].

Citrus flavonoids enhance BBB integrity by upregulating tight junction proteins such as occludin and claudin-5 while inhibiting matrix metalloproteinases (MMPs) that degrade the vascular barrier [76]. Hesperidin has been shown to increase claudin-5 expression in LPS-exposed endothelial cells, thereby reducing vascular permeability and neuroinflammatory damage [121]. Furthermore, metabolites like naringenin-7-O-glucuronide cross the BBB via glucose transporters (GLUT1), achieving brain concentrations sufficient for neuroprotection despite the inherently low systemic bioavailability of flavanones [78,122].

Despite promising preclinical findings, the limited bioavailability of citrus polyphenols remains a challenge, necessitating advanced strategies such as nanoformulations to enhance BBB penetration and improve compound stability during digestion [120,123].

**Figure 4:**
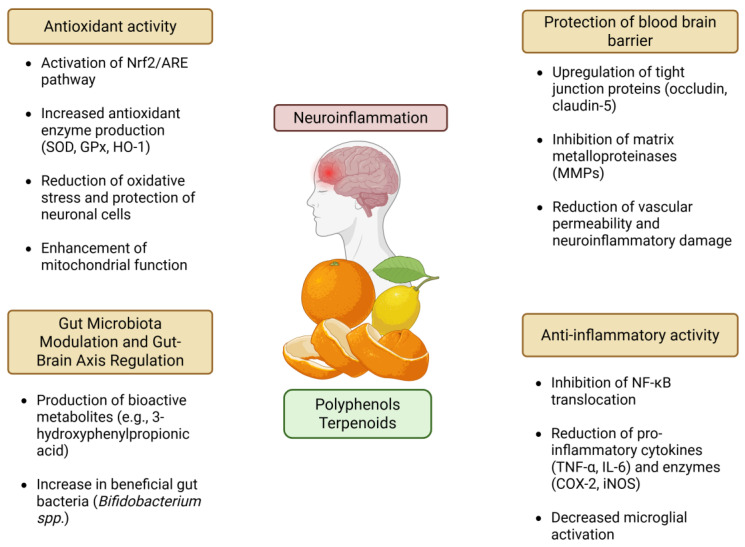
Anti-neuroinflammatory activities of citrus waste-derived active compounds. The figure is original and created with BioRender.com (accessed on 4 April 2025).

Additionally, individual variations in gut microbiota composition significantly influence the metabolic efficacy of flavanones, suggesting that personalized approaches may optimize their therapeutic potential [124]. Large-scale human trials are essential to validate the long-term safety and clinical efficacy of citrus polyphenols across diverse populations. These compounds offer a multifaceted neuroprotective approach through key mechanisms, including antioxidant defense via Nrf2 activation, anti-inflammatory modulation through NF-κB inhibition, gut–brain axis regulation, BBB stabilization, mitochondrial protection, and neurotransmitter modulation, as depicted in Figure 4. Beyond their health benefits, the valorization of citrus polyphenols provides a sustainable strategy for repurposing agricultural by-products, paving the way for innovative nutraceutical interventions to prevent or manage neurodegenerative diseases such as AD and PD. Table 1 summarizes the main molecular pathways modulated by these citrus-derived compounds, their biological effects, and corresponding references.

## 6. Conclusions and Future Directions

Citrus by-products have emerged as a promising source of bioactive compounds with potential therapeutic applications, particularly in the context of neuroinflammation. As a key pathological mechanism underlying the development and progression of neurodegenerative diseases such as AD, PD, and multiple sclerosis, neuroinflammation represents a critical target for intervention. The anti-inflammatory and antioxidant properties of various phytochemicals present in citrus waste underscore its relevance as a valuable yet often overlooked resource for developing novel neuroprotective strategies.

A key avenue in the application of citrus waste in neuroinflammation lies in the investigation of flavonoids, which are particularly abundant in citrus peels.

Among these compounds, hesperetin, naringenin, nobiletin, and rutin have emerged as the most promising candidates due to their ability to mitigate oxidative stress and reduce neuroinflammatory markers. These effects are primarily mediated through the activation of the Nrf2/ARE antioxidant pathway and the inhibition of NF-κB signaling, both of which are critical to the regulation of neuroinflammation. Furthermore, the capacity of these compounds to cross the blood–brain barrier, regulate mitochondrial and cellular stress responses, and influence autophagy-related pathways further supports their potential as nutraceutical agents. In parallel, their metabolites play a significant role in maintaining gut–brain axis homeostasis by promoting the production of SCFA, thereby enhancing their anti-neuroinflammatory properties through both systemic and central mechanisms. Future research may aim to further elucidate the specific molecular mechanisms underlying these effects, with particular attention to their modulation of microglial activation.

Moreover, optimizing the bioavailability and delivery mechanisms of citrus waste bioactive molecules will be crucial. Since many of the bioactive compounds are poorly absorbed in their natural form, nanotechnology, liposomal encapsulation, or other advanced drug delivery systems could be developed to enhance their therapeutic potential in treating neuroinflammation.

Notably, utilizing citrus-derived extracts instead of isolated compounds presents an appealing strategy due to the potential synergistic interactions amongst their various bioactive constituents. These interactions may enhance therapeutic efficacy and expand the spectrum of neuroprotective effects. However, it is crucial to recognize that the phytochemical composition of plant extracts can be influenced by factors such as geographic origin, cultivation conditions, and extraction methodologies, which may impact reproducibility. Although isolated compounds facilitate the creation of more standardized formulations, they often lack the complexity and multi-target actions found in the whole extracts and may incur higher production costs. In this regard, optimizing standardized extraction protocols could mitigate these challenges and promote the integration of citrus waste extracts into evidence-based strategies for the management of neuroinflammation.

In conclusion, the future of citrus waste in neuroinflammation is promising, supported by growing evidence of its potential as an adjunctive treatment. Advancements in the characterization of bioactive constituents, optimization of delivery systems, and elucidation of molecular pathways may pave the way for the development of citrus by-products as promising therapeutic candidates for the management of neuroinflammatory disorders.

## Figures and Tables

**Table 1:** Overview of bioactive compounds derived from *Citrus* species, their molecular targets and pathways, associated biological activities, and key literature references.

Compound	Citrus Source	Target Gene/Pathway	Biological Activity	Ref.
Hesperidin	*C. sinensis* *C. reticulata* *C. grandis* *C. limon*	↑ HO-1, SOD, CAT, GSH ↓ iNOS, COX-2, PGE_2_, NO ↓ ICAM-1, VCAM-1, IL-1β, IL-6, IL-17 and TNF-α ↑ IL-10, TGF-B ⊣ PDEs ↑ Claudin-5 ↑ AMPK ↓ mTOR ↓ Caspase-3 ↓ APP, Aβ1–42 ↓ AChE activity	Antioxidant activity, ROS scavenging activity, anti-inflammatory activity, vascular permeability activity and BBB protection, anti-apoptotic activity, gut microbiota modulation, reduction in Aβ deposits, autophagy-promoting activity.	[4,7,8,51,52,54,63,64,76,81,91,102,105,117,118]
Hesperetin	*C. sinensis* *C. reticulata* *C. grandis* *C. limon*	↑ Nrf2/ARE ↑ HO-1, SOD, CAT, GSH ↓ NF-κB ↓ TNF-α, VCAM-1, IL-17, IL-6 and IL-1β. **↑** IL-10, TGF-β ↓ iNOS, COX2 ↓ Caspase-3, Bax, ↑ Bcl-2 ↑ PI3K/Akt, ERK/MAPK **↑** Th2/Treg	Antioxidant activity, ROS scavenging activity, anti-inflammatory activity, anti-apoptotic activity, apoptosis reduction in PD models, reduction in CNS demyelination, immune cell modulation.	[54,76,90,91,93,95,102,105]
Naringin	*C. sinensis* *C. limon* *C. paradisi* *C. reticulata* *C. aurantium* *C. grandis*	↑ SOD-1, CAT, GSH ↓ MDA ↓ LPO ↓ NF-κB, ↓ MAPK (p38) ↓ IL-6, TNF-α, IL-1β, MCP-1, VCAM-1, ICAM-1 ↓ iNOS, NO, ↓ Caspase-3, Bax ↓ GRP78 ↓ CHOP ↓ ATF6 ⊣ PDEs ⊣ TXA_2_ formation	Antioxidant activity, ROS scavenging activity, anti-inflammatory activity, anti-apoptotic activity, ER stress-reducing activity, vasoprotective activity, cognitive-enhancing activity, cytoprotective (H_2_O_2_ damage).	[7,8,51,52,53,54,59,63,76,86,89,102,109]
Naringenin	*C. sinensis* *C. limon* *C. paradisi* *C. reticulata* *C. grandis*	↑ Nrf2/ARE ↑ SOD-1, CAT, GSH ↓ MDA ↓ iNOS, COX2 ↓ NF-κB, ↓ MAPK (JNK, p38), ↓ STAT-1, ↑ SOCS3 ⊣ TLR4/NF-κB, ↓ Iba-1, GFAP ↓ IL-1β, IL-17, IL-6, TNF-α, MCP-1, VCAM-1 ↓ BACE1 ↓ Caspase-3, Bax ↑ Bcl-2 ↑ AMPKα ↓ AChE/BChE ↓ GSK-3β activity ↑ ACh ↑ BDNF, NGF ↑ AMPK/ULK1 axis ↓ mTOR ↑ PI3K/Akt, MAPK/ERK ⊣ UV-induced damage	Antioxidant activity, ROS scavenging activity, anti-inflammatory activity, anti-amyloidogenic activity, anti-apoptotic activity, cognitive-enhancing activity, autophagy-promoting activity, cytoprotective activity (UV damage).	[7,52,54,76,77,78,81,85,102,111,118,122]
Nobiletin	*C. reticulata* *C. sinensis* *C. tangerina*	↑ Nrf2/ARE ↑ SOD-1, CAT, GSH ↓ LOP, ↓ MDA, ↓ iNOS, COX-2 ↓ GSSG ↓ MMP-9 ↓ NF-κB, ↓ MAPK (JNK, p38), ↓ TNF-α, IL-1α, IL-17, IL-1β, IL-6, PGE_2_ **↓** proMMP-1/proMMP-3 ↑ PI3K/Akt, MAPK/ERK ↓ NO ⊣ BACE1 ↓ Caspase-3, Bax, ↑ Bcl-2 ↑ CREB-P ↑ mRNA NR2B, NR2A, NR1, ChAT, mAChR M1	Antioxidant activity, ROS scavenging activity, anti-inflammatory activity, anti-angiogenic activities, enhancement in glutamatergic and cholinergic neurotransmission, anti-apoptotic activity, improvement in synaptic plasticity, anti-amyloidogenic activity, cognitive-enhancing activity.	[4,51,52,54,56,59,64,81,110,118,119]
Eriocitrin	*C. limon* *C. reticulata* *C. bergamia* *C. aurantium*	↑ Nrf2 ↑ SOD, HO-1 ↑ NQO1 ↓ MDA ↓ NF-κB p65 ↓ TNF-α, IL-6 ↑ IL-10 ↓Caspase-3, caspase-9 ↑AMPK ↓ mTOR	Antioxidant activity, anti-inflammatory activity, anti-apoptotic activity, autophagy inducer.	[54,65,76,91,94]
Rutin	*C. aurantium* *C. reticulata* *C. grandis* *C. sinensis*	↑ CAT ⊣ TLR9/NF-κB axis ↓ Alox4a, Alox5 ↓ RelA, ↓ NOS2a ↓ TNF-α, IL-6, IL-1β, CXCL8 ↓ MIF ↓ AChE activity ↓ prnpa, itgb2, ALP	Antioxidant activity, ROS scavenger activity, anti-inflammatory activity, reduction in immune cell infiltration, neuroprotective, and anti-neuroinflammatory activity.	[53,63,85,88]
Narirutin	*C. reticulata* *C. sinensis*	↑ SOD, CAT, GSH, and HO-1 ↓ TNFα, IL-6, and IL-1β ↓ NF-kB ⊣ AChE ↑ IκBα ⊣ JAK2/STAT3	Antioxidant, activity, oxidative stress reduction, anti-inflammatory activity, synaptic plasticity improvement, neuronal apoptosis reduction, memory and learning improvement, attenuates systemic and cerebral inflammation, cognitive decline prevention.	[8,54,73,83]
Tangeretin	*C. reticulata* *C. sinensis*	↓ TNFα, IL-6, Il-2, and IL-1β ↑ SOD-1, CAT, HO-1 ↓ iNOS, COX-2 ↑ Nrf2/HO-1 ↓ NF-kB ↑ PI3K/Akt ⊣ AChE ↑ IκBα	Anti-inflammatory activity, oxidative stress reduction, neural cell death reduction, neurogenesis increase, cognition and memory improvement, neurodegeneration reduction, mitigated neurological abnormalities and acute brain injury mitigation, synaptic impairment reduction, Aβ aggregation inhibition.	[4,51,52,56,59,64,81,104,107,110]
Quercetin	*C. reticulata* *C. sinensis*	↑ Nrf2/ARE ⊣ AChE and BChE ↑ SOD-1, CAT, GSH, and GPx1 ↓ TNFα, IL-6, and IL-1β ↓ NF-kB **↓** BACE1 ⊣ Iba-1, and GFAP ↑ ATP synthesis	Antioxidant activity, oxidative stress reduction, ROS/RNS scavenger activity, anti-inflammatory activity, neuron protection, anti-amyloidogenic properties, Aβ aggregation inhibition, mood, motor, memory deficits, and learning function improvement, tau phosphorylation reduction, inhibition of platelet aggregation, cognitive enhancement, mitochondrial dysfunction modulation.	[54,59,60,63,85,106,118]
Limonene	*C. limon* *C. sinensis* *C. aurantium*	**↓** TNF-α, IL-6, and IL-1β ⊣ AChE and BChE ⊣ LOX	Antioxidant activity, ROS/RNS scavenger activity, anti-inflammatory capacity, oxidative stress protection, reduction in Aβ deposits.	[52,61,62,81]
Limonin	*Citrus* spp.	↑ TPH	Neuroprotective effects, anti-apoptotic activity, amino acid content upregulation.	[103]
Phenolic acids (e.g., gallic acid, ferulic acid, and caffeic acid)	*Citrus* spp.	⊣ BACE1 activity ↓ TNFα, and IL-1β ↑ SOD-1, CAT, and GPx1 ↓ GFAP ↑ Nrf2/HO-1	Antioxidant activity, ROS scavenger activity, anti-apoptotic activity, microglial inhibition activation, reduction in Aβ deposits, improvement in spatial cognitive and memory functions, neuroinflammation attenuation, synaptic strength increasing.	[53,63,64,81,84,118]
Pectin	*Citrus* spp.	↑ MAPK/ERK ↓ PI3K/Akt ↓ NF-kB	Antioxidant activity, ROS scavenger activity, neuroinflammatory response inhibition, microglial inhibition activation, anti-apoptotic activity.	[81,91,92]

**Note:** Symbols ↑, ↓, ⊣ indicate induction/up-regulation, reduction/down-regulation, inhibition, respectively. **Abbreviations**: Aβ1–42: Amyloid-beta peptide 1-42; ACh: Acetylcholine; AChE: Acetylcholinesterase; ALP: Alkaline phosphatase; Alox4a: Arachidonic acid 4 alpha-lipoxygenase; Alox5: Arachidonate 5-lipoxygenase a; AMPK: AMP-activated protein kinase; AMPKα: AMPK alpha subunit; APP: Amyloid Precursor Protein; ARE: Antioxidant response element; ATF6: Activating transcription factor 6; ATP: Adenosine triphosphate; BACE1: Beta-secretase 1; Bax: Bcl-2-associated X protein; Bcl-2: B-cell lymphoma 2; BChE: Butyrylcholinesterase; BDNF: Brain-derived neurotrophic factor; C/EBP: CCAAT/enhancer-binding protein; CAT: Catalase; ChAT: Choline acetyltransferase; CHOP: C/EBP homologous protein; COX-2: Cyclooxygenase-2; CREB-P: Phosphorylated cAMP response element-binding protein; CXCL8: Chemokine (C-X-C motif) ligand 8; ERK/MAPK: Extracellular signal-regulated kinase/Mitogen-activated protein kinase; GFAP: Glial fibrillary acidic protein; GPx1: Glutathione peroxidase 1; GRP78: Glucose-regulated protein 78 kDa; GSH: Reduced glutathione; GSSG: Oxidized glutathione; HO-1: Heme oxygenase-1; Iba-1: ionized calcium-binding adapter molecule 1; ICAM-1: Intercellular Adhesion Molecule-1; iNOS: inducible Nitric Oxide Synthase; IL-10: Interleukin-10; IL-1α: Interleukin-1 alpha; IL-1β: Interleukin-1 beta; IL-6: Interleukin-6; IL-17: Interleukin-17; itgb2: integrin beta-2; IκBα: Inhibitor of nuclear factor kappa B alpha; JAK2/STAT3: Janus kinase 2/Signal transducer and activator of transcription 3; JNK: c-Jun N-terminal kinase; LOP: Lipid oxidation products; LOX: Lipoxidase; LPO: Lipid peroxidation; mAChR M1: muscarinic acetylcholine receptor M1; MAPK: Mitogen-activated protein kinase; MCP-1: Monocyte chemoattractant protein-1; MDA: Malondialdehyde; MIF: Macrophage migration inhibitory factor; MMP-9: Matrix metalloproteinase-9; mTOR: mammalian target of rapamycin; NF-κB: Nuclear factor kappa-light-chain-enhancer of activated B cells; NGF: Nerve growth factor; NO: Nitric Oxide; NQO1: NAD(P)H quinone dehydrogenase 1; NR1: N-methyl-D-aspartate receptor subunit 1; NR2A: N-methyl-D-aspartate receptor subunit 2A; NR2B: N-methyl-D-aspartate receptor subunit 2B; Nrf2: Nuclear factor erythroid 2-related factor 2; PDEs: Phosphodiesterases; PGE_2_: Prostaglandin E_2_; PI3K/Akt: Phosphatidylinositol 3-kinase/Protein kinase B; prnpa: prion protein a; proMMP-1: Pro-matrix metalloproteinase-1; proMMP-3: Pro-matrix metalloproteinase-3; RelA: RELA protooncogene; SOCS3: Suppressor of cytokine signaling 3; SOD: Superoxide Dismutase; SOD-1: Superoxide Dismutase 1; STAT-1: Signal transducer and activator of transcription 1; Th2: T helper type 2 cells; TGF-β: Transforming Growth Factor beta; TXA_2_: Thromboxane A_2_; TLR4: Toll-like receptor 4; TLR9: Toll-like receptor 9; TNF-α: Tumor Necrosis Factor alpha; TPH: Tryptophan hydroxylase; Treg: Regulatory T cells; ULK1: Unc-51 like autophagy activating kinase 1; UV: Ultraviolet; VCAM-1: Vascular Cell Adhesion Molecule-1.
